# Inflammatory markers and mediators in heart disease

**DOI:** 10.18632/aging.101640

**Published:** 2018-11-09

**Authors:** Ana M. Valdes, Cristina Menni

**Affiliations:** 1 School of Medicine, University of Nottingham, Nottingham, UK; 2 NIHR Nottingham Biomedical Research Centre, Nottingham, UK; 3 Department of Twin Research and Genetic Epidemiology, King's College London, London, UK

**Keywords:** inflammation, cardiovascular disease, gut microbiome, protein glycosylation

Inflammation is a key driver in aging and age-related diseases and is now known to be one of the main causes of heart disease, a major cause of death in Europe and the US [1]. In a meta-analysis of 29 population-based prospective studies, interleukin-6, interleukin-18, and the tumor necrosis factor alpha (IL-6, IL-18 and TNF-α) were all found to result in significantly higher relative risks for non-fatal myocardial infarction or coronary heart disease death after adjusting for traditional risk factors [1]. The role of modulating the inflammatory process as a therapeutic target has been clearly demonstrated by the CANTOS trial (Canakinumab Anti-Inflammatory Thrombosis Outcomes Study) [2] with respect to atherothrombosis development. Indeed, inhibiting interleukin-1β reduced the incidence of repetitive atherothrombotic events.

Recently, protein glycosylation- the enzymatic attachment of sugar molecules to proteins, has been strongly implicated in inflammation. Glycosylation is one of the most important and complex post-translational mechanisms regulating protein function, folding, localization, and stability [2]. One measure of glycosylated protein called GlycA, is associated with an increased risk of heart disease [2]. However, there are many different types of glycosylated proteins and overall they constitute the "glycome". Immunoglobulin G (IgG) occupies a central role in the immune system and the conserved N-glycan at asparagine 297 can modulate inflammatory responses. Indeed, studies have shown that a lack of certain sugars attached to this position increased the ability of IgG to induce antibody-dependent cell mediated cytotoxicity in mice [3]. Low galactosidation and sialylation at this site were also associated with viral infections and have been proposed as markers of chronic systemic low level inflammation in elderly individuals [3].

In this our recent paper, we measured a comprehensive set of these molecules to understand their involvement in the risk of atherosclerosis. We measured 76 glycosylated IgG measures or “glycans” plus GlycA in 845 men and 3937 women from two independent cohorts in the United Kingdom [4]. Six of the tested glycans were associated with the summary risk score of atherosclerotic disease. Four glycans, in addition to the previously reported GlycA, were also associated with measures of atherosclerosis after adjusting for the other known cardiovascular risk factors, such as cholesterol, smoking and blood pressure. Combining all the significant glycan factors gives incremental information on the presence of atherosclerosis, possibly though summation of the total amount of inflammation present in an individual, which contributes to their risk of heart disease.

Importantly, inflammation is also strongly associated with arterial stiffness [5] and the composition of the gut microbiome, the microbes that leave in the human colon, has now been widely accepted to be an important determinant of inflammatory status [5]. We have recently demonstrated that gut microbiome composition influences arterial stiffness, partly via its effects on insulin resistance, visceral fat and c-reactive protein (CRP) [6]. However, as the vast majority of the effect is not mediated by these mechanisms and given the strong links between low microbiome diversity and inflammatory diseases such as eczema, psoriasis and inflammatory bowel disease [5], we hypothesised that the effect of the gut microbiome composition on arterial stiffness is likely to be in large part caused by its modulation of systemic inflammation in other ways not reflected by CRP. If this proves to be indeed the case it opens the possibility of intervening therapeutically through the gut microbiome to reduce cardiovascular risk.

In our study, we reported that 8.4% of the variation in arterial stiffness is explained by gut microbiome composition [6]. To put this into context, a ~10% difference in measure of arterial stiffness is, for instance, what is reported in improvement of arterial stiffness after between pre and post kidney transplantation in patients with end-stage kidney disease [7]. Therefore it is a magnitude that is likely to be clinically significant and corresponds broadly to about 1 m/s. In the Framingham cohort, each additional m/s in arterial stiffness corresponds to an almost 50% increase in the longitudinal risk over 7 years (HR=1.48%) of developing a cardiovascular event [7].

**Figure 1:**
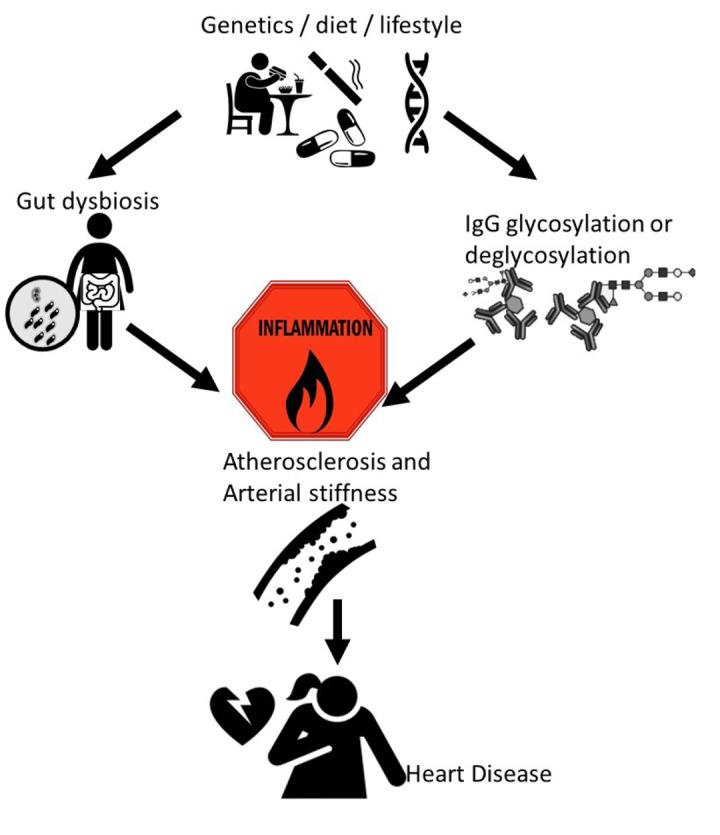
Our recent work linking inflammatory markers and mediators to subclinical atherosclerosis and arterial stiffness, both of which are early stages of heart disease.

These results relating to biomarkers and modulators of inflammation in heart disease are, at this early stage, unlikely to change clinical practice. However, for example, the recommendation for more dietary fibre intake is already part of prevention guidelines, as there is strong evidence that higher dietary fibre intake protects from the development of cardiovascular events [5]. Establishing the link between nutritional or lifestyle factors and mechanisms related to inflammation in cardiovascular risk can help us device better interventions to prevent or delay cardiovascular disease (Figure 1).
